# Network analysis of intimate partner violence and depression–anxiety symptoms in Chinese transgender women

**DOI:** 10.1186/s12889-026-28745-8

**Published:** 2026-07-27

**Authors:** Lulu Xu, Yawen Zheng, Fengyi Wang, Jiani Hu, Fang Wang, Ying Wang, Yong Cai

**Affiliations:** 1 Institute of Clinical Research, Tongren Hospital, Shanghai Jiao Tong University School of Medicine, 1111, Xianxia Road, Shanghai, 200336 China; 2 School of Public Health, Shanghai Jiao Tong University School of Medicine, 227 South Chongqing Road, Shanghai, 200025 China; 3 Department of Student Affairs, Shanghai Jiao Tong University School of Medicine, 227, South Chongqing Road, Shanghai, 200025 China; 4 Youth Science and Technology Innovation Studio, affiliated with Tongren Hospital, Shanghai Jiao Tong University School of Medicine, 1111 Xianxia Road, Shanghai, 200335 China

**Keywords:** Chinese transgender women, Intimate partner violence, Depression, Anxiety, Network analysis

## Abstract

**Background:**

Chinese transgender women have a higher prevalence of intimate partner violence (IPV) compared to their cisgender counterparts. To mitigate the risk of IPV, it is crucial to comprehend the symptoms of emotional distress that are most closely associated with IPV. This network analysis investigated the item-level connections among depressive symptoms, anxiety symptoms, and IPV among Chinese transgender women.

**Methods:**

Between February and December 2022, 264 adult transgender women in Shenyang, China, were recruited through snowball sampling with support from a community-based non-governmental organization. Data were collected using the Intimate Partner Violence Scale, the Patient Health Questionnaire (PHQ-9), and the Generalized Anxiety Disorder Questionnaire (GAD-7). The network structure of depressive symptoms, anxiety symptoms, and IPV was examined using “Expected Influence” and “Bridge Expected Influence” as measures of centrality. The stability of the network was assessed through a case-dropping bootstrap approach. A Network Comparison Test was conducted to examine whether network characteristics differed based on the presence or absence of IPV.

**Results:**

Among 264 participants, 221 (83.7%) reported having experienced IPV. The most central symptom was PHQ6 (guilt), followed by GAD2 (uncontrollable worry) and GAD1 (nervousness). The bridge symptoms linking the depression and anxiety communities were GAD2 (uncontrollable worry), PHQ9 (suicide), and GAD5 (restlessness). A flow network indicated that the strongest associations between IPV and depressive and anxiety symptoms were for GAD5 (restlessness) and PHQ2 (sad mood). Network Comparison Test revealed no significant differences in global strength or structure between the IPV and non-IPV groups.

**Conclusions:**

Findings highlight specific depressive and anxiety symptoms as key correlates of IPV among Chinese transgender women. These central and bridge symptoms offer hypothesis-generating entry points for future longitudinal research and may inform the development of targeted interventions; however, the cross-sectional design precludes causal or directional conclusions.

**Supplementary Information:**

The online version contains supplementary material available at https://doi.org/10.1186/s12889-026-28745-8.

## Background

Intimate partner violence (IPV), defined as actions carried out by a current or former intimate partner that lead to physical, sexual, or psychological harm [1], represents a substantial public health issue of epidemic proportions, impacting individuals globally irrespective of gender or socioeconomic status. A recent secondary analysis of population-based surveys indicates that the incidence of IPV against women aged 15 to 49 has persisted at a high level, revealing that 37.2% of women in this age group across 53 low and middle-income countries experienced such violence between 2000 and 2021 [2].

To date, the bulk of research on IPV has focused on cisgender women in heterosexual relationships, despite evidence suggesting a higher prevalence of IPV among transgender women [3]—individuals assigned male at birth but who identify as female. Studies show that 37.5%-57% of transgender individuals experience IPV, which is 1.7 times the rate among cisgender individuals. They are also 2.2 times more likely to suffer physical IPV and 2.5 times more likely to face sexual IPV [4–7]. Critically, beyond this elevated overall prevalence, transgender women bear an additional and unique burden of IPV directly related to their transgender identity—a form of victimization that cisgender women do not typically encounter. For example, a study estimated that young transgender women survivors faced various types of IPV, including controlling behaviors (67.9%), physical threats (47.4%), sexual abuse (38.5%), as well as identity-specific IPV such as being coerced into gender-incongruent activities (42%) and experiencing psychological abuse based on their gender identity (53%) [8]. This distinct category of identity-based violence, which targets the victim’s fundamental sense of self, represents a unique form of IPV experienced specifically by transgender individuals and contributes significantly to their greater risk of IPV compared to their cisgender counterparts.

Previous studies have found bidirectional relationships between IPV [9, 10] and psychiatric symptoms [11–13]. For example, one study found that IPV is associated with mental health problems including anxiety and depression among transgender and gender nonconforming adults, all four types of IPV were positively associated with anxiety, and all but physical abuse were significantly associated with depression [14]. A further study involving Black transgender women found that when recent IPV, defined as experiences within the past 12 months, was treated as a covariate, it reduced the correlation between social gender affirmation and mental health outcomes to a non-significant association. This suggests that IPV exposure may have a temporal impact on mental health, potentially overshadowing any beneficial effects of social gender affirmation [15].

Psychiatric syndromes, such as depression, have traditionally been viewed as overt manifestations of a singular underlying condition. This conventional perspective, however, overlooks the causal factors, the evolution of symptoms, their diversity, and the intricate interconnections among various symptoms [16, 17]. Consequently, the inherent complexity of mental health issues is frequently simplified, as symptoms are addressed merely as visible, uniformly significant indicators of a unified disorder [16, 18]. Network analysis approaches represent innovative statistical methodologies that conceptualize mental health issues as interrelated systems of symptoms. These symptoms can potentially engender one another, manifesting as byproducts or epiphenomena throughout the development towards a disorder [19]. In network theory, central nodes are the most influential indicators of a disorder, capable of triggering other symptoms and playing a pivotal role in the initiation and/or perpetuation of a syndrome. Network analysis holds the potential to delineate specific relationships between individual symptoms of a disorder and identify symptoms that may warrant further clinical attention and investigation [20]. Furthermore, network analysis proves to be an effective tool for discerning the structure of psychiatric disturbances from clinical data [19, 21]. It also serves to illuminate significant connections among individual symptoms, whether within a single disorder or across various conditions [22].

Little previous research using network analysis has been conducted to clarify the interrelations between IPV and psychiatric symptoms among Chinese transgender women. One study conducted in the Chinese context examined the impact of parental psychological abuse on the psychopathological symptoms of gender minority youth. That study found that, within the subgroup of transgender women, the most influential core symptoms in the depression network model included “effort”, “sadness”, “control worry”, and “depressed mood” [23]. Although that study did not directly examine IPV, its findings demonstrate that network analysis is feasible for identifying core depressive symptoms in this population. Given that anxiety frequently co-occurs with depression [24] and may be independently associated with IPV [14], future network analysis studies that directly focus on IPV—and that incorporate both depressive and anxiety symptoms—could help clarify the specific dimensions of emotional distress most relevant to IPV among Chinese transgender women.

To better understand the mental health correlates of IPV exposure in this vulnerable population, it is important to examine how specific depressive and anxiety symptoms are associated with IPV. Therefore, this study used network analysis to explore item-level associations among depressive symptoms, anxiety symptoms, and IPV exposure among Chinese transgender women. Unlike studies relying only on total depression or anxiety scores, this approach may help identify symptoms that are statistically central within the depression–anxiety network and symptoms most strongly associated with IPV exposure. Because the data are cross-sectional, the study was intended to generate hypotheses rather than establish causal pathways or intervention effects.

## Methods

### Participants

Two hundred and sixty-four Chinese transgender women were recruited in Shenyang, China, from February to December 2022. Inclusion criteria encompassed individuals aged 18 or above, self-identifying as transgender women, residing and working in Shenyang, and expressing willingness to participate in the study following an elucidation of the study objectives with an assurance of confidentiality. Exclusions were made for those with evident severe mental illness, psychological conditions, intellectual disabilities, or an inability to collaborate in survey completion. All participants were informed of the guarantee of anonymity and the right to withdraw at any time. They were also assured that no consequence for refusing the survey. Demographic information is presented in Table 1.

**Table 1:** Socio-demographic characteristics of Chinese transgender women and overall and subgroup associations with intimate partner violence (*N* = 264)

Panel A. Overall IPV exposure and IPV subtype prevalence						
Variables	Number of participants N (%)	IPV Mean (*SD*)			IPV Yes N(%)	
Total IPV	264(100.0)	10.24(3.65)			221(83.7)	
Experienced psychological IPV	264(100.0)	2.17(0.86)			203(76.9)	
Experienced physical IPV	264(100.0)	2.08(0.96)			176(66.7)	
Experienced sexual IPV	264(100.0)	1.95(0.95)			159(60.2)	
Experienced transgender-specific forms of IPV	264(100.0)	4.04(1.58)			202(76.5)	

Panel B. Sociodemographic characteristics and associations with IPV						
Variables	Number of participants N (%)	IPV Mean (*SD*)	*F*	*P*	IPV Yes N(%)	OR(95%CI)
Age(mean ± *SD*)	264 (29.99 ± 6.38)	—	—	—	—	—
Sexual orientation n (%)			2.085	0.103		
Heterosexuality	67(25.4)	9.61(4.24)			51(23.1)	1
Homosexuality	129(48.9)	10.71(3.37)			112(50.7)	2.067(0.968 − 4.414)
Bisexuality	52(19.7)	10.27(3.51)			46(20.8)	2.405(0.868 − 6.666)
Others	16(6.1)	8.94(3.30)			12(5.4)	0.941(0.266 − 3.329)
Educational level n (%)			0.822	0.483		
Primary school and below	16(6.1)	9.56(3.16)			12(5.4)	1
Middle school	65(24.6)	10.26(4.31)			51(23.1)	1.214(0.339 − 4.353)
High school/vocational school	101(38.3)	10.62(3.51)			90(40.7)	2.727(0.748 − 9.939)
College degree or above	82(31.1)	9.88(3.35)			68(30.8)	1.619(0.455 − 5.762)
Marital status n (%)			2.376	0.070		
Married	23(8.7)	9.13(3.82)			19(8.6)	1
Unmarried	203(76.9)	10.16(3.69)			169(76.5)	1.046(0.335 − 3.270)
Divorced	35(13.3)	11.54(3.08)			31(14.0)	1.632(0.364 − 7.305)
Widowhood	3(1.1)	9.00(3.46)			2(0.9)	0.421(0.030 − 5.850)
Monthly income n (%)			**6.160** ^***^	**< 0.001**		
3000 RMB and below	77(29.2)	8.96(3.56)			57(25.8)	1
3001–6000 RMB	87(33.0)	10.18(3.66)			71(32.1)	1.557(0.740 − 3.277)
6001–12,000 RMB	75(28.4)	11.32(3.28)			69(31.2)	**4.035(1.518** − 10.724)^**^
12,001 RMB and above	25(9.5)	11.12(3.92)			24(10.9)	**8.421(1.069** − 66.350)^*^
Length of residence in Shenyang n (%)			2.245	0.084		
Local residents	123(46.6)	9.88(3.93)			96(43.4)	1
1 year and below	26(9.8)	10.77(3.67)			23(10.4)	2.156(0.602 − 7.729)
1–5 years	65(24.6)	11.12(2.99)			59(26.7)	**2.766(1.078** − 7.095)^*^
5 years and above	50(18.9)	9.70(3.58)			43(19.5)	1.728(0.698 − 4.274)
Working condition n (%)			2.261	0.106		
Employed	96(36.4)	9.92(3.45)			80(36.2)	1
Unemployed	144(54.5)	10.63(3.73)			124(56.1)	1.240(0.607 − 2.535)
Others	24(9.1)	9.17(3.78)			17(7.7)	0.486(0.173 − 1.362)
Smoking status n (%)			**7.967** ^**^	**0.005**		
No	84(31.8)	9.32(3.41)			68(30.8)	1
Yes	180(68.2)	10.67(3.70)			153(69.2)	1.333(0.675 − 2.635)
Drinking status n (%)			**8.448** ^**^	**0.004**		
No	60(22.7)	9.05(3.98)			42(19.0)	1
Yes	204(77.3)	10.59(3.48)			179(81.0)	**3.069(1.535** − 6.135)^**^
Drugs use status n (%)			0.031	0.861		
No	250(94.7)	10.25(3.69)			209(94.6)	1
Yes	14(5.3)	10.07(2.95)			12(5.4)	1.177(0.254 − 5.457)

Odds ratios were estimated from univariate logistic regression models using dichotomous lifetime IPV exposure as the outcome

*IPV*  Intimate partner violence

^*^ *P* < 0.05, ^**^ *P* < 0.01, ^***^ *P* < 0.001

### Procedures

Because transgender women in China are hard to reach and hidden due to pervasive discrimination, snowball sampling was used. Participants were recruited through a local non-governmental organization (NGO) called China Love Aid, whose mission to improve transgender women’s physiological and psychological health made it a trusted gateway into this population. The NGO staff, who offer services to transgender women, reached out to their entire clientele to facilitate recruitment. With the NGO’s aid, five transgender women were enlisted as “seeds”. These “seeds” subsequently recruited or referred additional eligible individuals, and the questionnaire survey was disseminated through this network until recruitment saturation was achieved (i.e., when no new participants could be identified). The NGO leaders also verified the saturation point, collaborating with local transgender women who were familiar with the local community and confirmed that recruitment had exhausted all possible contacts. To maintain high data quality, sufficient validation checks were applied, including the verification of participant eligibility by NGO staff and trusted “seeds”, as well as the screening of incomplete or duplicate submissions during data cleaning.

Before their participation, all participants gave their written informed consent. Upon entering the survey, participants were first asked to provide demographic information (e.g., age, sexual orientation, income). They then completed measures assessing depression, anxiety and IPV. The measures were presented in this sequence because starting with a sensitive topic such as IPV might discourage participants from completing the survey. Finally, upon submission of the questionnaire, participants received a 200 RMB (approximately 30 USD) incentive.

### Measures

#### Background characteristics

The following background characteristics were collected: age, sexual orientation, educational level, marital status, monthly income, length of residence in Shenyang, working condition, smoking status, drinking status, and drugs use status. Age was analyzed as a continuous variable. Other variables were treated as categorical variables (Table 1). Duration of residence in Shenyang was specifically included because it may reflect social integration, stability of local networks, access to community resources, and migration-related vulnerability.

#### Anxiety

Anxiety symptoms were measured with the Generalized Anxiety Disorder Questionnaire (GAD-7), developed by Spitzer et al. [25]. The GAD-7 includes seven self-report items that are scored on a 4-point Likert scale based on symptom frequency over the past two weeks: 0 = “Not at all”, 1 = “Several days”, 2 = “More than half the days”, 3 = “Nearly every day”. Higher total scores indicate greater severity of anxiety symptoms. The Chinese version of the GAD-7 has been widely used and demonstrates adequate reliability and validity [26]. In our study, the GAD-7 demonstrated high internal consistency (Cronbach’s α = 0.915).

#### Depression

Depression among transgender women was assessed using the Patient Health Questionnaire (PHQ-9) [27, 28]. The PHQ-9 includes nine self-report items scored on a 4-point Likert scale based on symptom frequency over the past two weeks: 0 = “Not at all”, 1 = “Several days”, 2 = “More than half the days”, and 3 = “Almost every day”. Higher total scores indicate greater severity of depressive symptoms. The Chinese version of the PHQ-9 has been widely used and demonstrates adequate reliability and validity [27]. In our study, the PHQ-9 demonstrated high internal consistency (Cronbach’s α = 0.915).

For descriptive comparisons between IPV and non-IPV groups (Table 2), total sum scores were calculated for both measures by adding responses across all items, with possible ranges of 0–21 for the GAD-7 and 0–27 for the PHQ-9. Higher total scores indicate greater severity of anxiety and depressive symptoms, respectively.

**Table 2:** Comparison of mental health among IPV and non-IPV groups

Symptoms	IPV group			Non-IPV group			Mean Range	T value	*P* value
	*N*	Mean	SD	*N*	Mean	SD			
Anxiety	221	9.06	4.67	43	2.65	4.11	6.41	−8.40	**< 0.001**
Depression	221	11.91	5.56	43	4.56	5.89	7.35	−7.86	**< 0.001**

However, for the primary network analysis, individual items from the GAD-7 and PHQ-9 were treated as separate nodes. No summation, averaging, or total scoring was applied to either scale. This approach preserves the unique variance of each symptom and allows for the examination of item-level connections rather than dimension-level associations.

#### Intimate partner violence

Intimate partner violence was assessed using a five-item scale developed for the Transgender Youth Research Project [8], assessing lifetime experiences of physical, psychological, sexual, and transgender-specific IPV perpetrated by a sexual partner. Response categories ranged from 1 (Never) to 4 (Many times). The total score ranges from 5 to 20. The scale demonstrated good internal consistency in the present study, with a Cronbach’s alpha of 0.858.

Participants were classified as IPV group if they reported a score of 2 or higher on any of the five items (indicating that the specific type of violence occurred at least “once” in their lifetime), and non-IPV group if they scored 1 (Never) on all items. This inclusive threshold was chosen to maximize sensitivity in capturing the broad spectrum of victimization—ranging from psychological coercion to identity-specific abuse—which is often underreported in this marginalized population. However, this approach prioritizes the presence of violence over its frequency or severity.

### Statistical analysis

#### Demographic data analysis

The data analyses were conducted using SPSS V.26 software. No missing data were observed for the primary variables of interest, as the electronic survey platform required all items including sociodemographic characteristics (e.g., age, monthly income, and educational level) and the primary scales (PHQ-9, GAD-7, and IPV) to be answered before submission. Continuous variables were reported as mean ± standard deviation, and categorical variables were expressed as numbers (percentages).

IPV was coded in two forms: a continuous summative score (a continuous summative severity score calculated by summing the five IPV item scores, with higher scores indicating greater cumulative IPV burden) and a dichotomous categorical variable (0 = no IPV, 1 = any IPV). The continuous measure was analyzed using univariate general linear models to detect linear relationships with socio-demographic factors, while the categorical measure was analyzed using logistic regression to compare any IPV exposure versus none. Both forms were used because they capture complementary aspects of IPV experience—cumulative burden and any victimization, respectively—each of which may have distinct socio-demographic correlates. *P*-values < 0.05 were considered statistically significant.

#### Network estimation

R (V.4.1.2) was used for data analysis and graphical visualization. Gaussian graphical models (GGMs) were used to establish the network structure among symptoms of depression and anxiety [29, 30]. GGM is an undirected network where nodes represent symptoms and edges represent the partial correlation coefficient between two symptoms in the network. GGM was selected because it estimates conditional associations between variables after controlling for all other variables in the network, which is particularly suited for identifying unique symptom-level relationships—such as central symptoms (most strongly connected to others) and bridge symptoms (connecting depression and anxiety communities)—and for isolating the specific associations between IPV and individual symptoms from confounding influences of other symptoms. To account for the ordinal nature and non-normal distribution of the symptom scores, the GGM was estimated using a non-parametric Spearman correlation matrix and regularized using the EBICglasso procedure. In the resulting regularized partial correlation network, edges represent the unique association between nodes after controlling for all remaining nodes in the network. Regularization shrinks small edge weights toward zero, reducing the likelihood of spurious associations and overfitting while improving network interpretability. In network analysis, nodes with stronger connections to other variables are positioned at the center, while those with weaker connections are placed on the periphery. In this model, blue edges represent positive associations and red edges represent negative associations, with thicker lines indicating stronger correlations. Consistent with the scale structures, two clusters were defined: one comprising the nine PHQ-9 depression items and the other comprising the seven GAD-7 anxiety items.

To examine associations with IPV while maintaining conceptual clarity, a flow network was estimated using the “flow” function in the R package “qgraph”, adjusting for selected sociodemographic and behavioral covariates (e.g., monthly income, length of local residence, smoking status, and drinking status). In this analysis, IPV was treated solely as an external binary exposure variable, not as a psychiatric symptom node within the depression–anxiety network. The flow network thus served to visualize partial correlations between IPV and each symptom, without implying that IPV occupies a symmetric position within the symptom network. IPV was positioned to the left, and depression and anxiety symptoms were positioned vertically to the right. Edges between IPV and symptoms represent direct or indirect partial correlations.

#### Centrality and stability

The expected influence (EI) was calculated using the centralityPlot function from the R package “qgraph”, which served to quantify the central symptom within the network [29]. A higher EI for a node signifies that it is a more central symptom within the network. The expected influence of the bridge (bEI), computed using the bridge function from the R package “networktools”, also serves to quantify the bridge symptoms that exist between the communities of depression and anxiety [31]. A node exhibiting a higher bEI between two mental disorders suggests a greater likelihood of these disorders being comorbid. Utilizing the R package “mgm”, the predictability of each node was computed; this metric indicates the extent to which a specific node can be accurately forecasted by all other nodes [32]. Predictability is depicted as a ring-shaped pie chart encircling each node within the network, with a more complete ring signifying greater predictability values. Network accuracy was evaluated using nonparametric bootstrap procedures by the R package “bootnet” [30], while stability was assessed using case-dropping bootstrap and the correlation stability coefficient (CS-coefficient). CS-coefficients above 0.25 are considered acceptable, whereas values above 0.50 indicate preferable stability. Node redundancy was assessed using Goldbricker (threshold = 0.25) from the R package “networktools”. All items were retained to maintain clinical granularity and diagnostic specificity.

#### Network comparison

The network structure was compared between the IPV and non-IPV groups using the R package “NetworkComparisonTest” (NCT), a permutation-based hypothesis test for network structure and global strength invariance [33]. The depression–anxiety symptom networks of these two groups were estimated and compared using the bootstrap method with 5,000 permutations at a significance level of 0.05.

## Results

### Characteristics of the participants

The demographic information and the differences in main variables are summarized in Table 1. A total of 264 Chinese transgender women participated in the study. The average age of the participants was 29.99 ± 6.38 years (range:18–53).

We first examined the continuous IPV severity score (the summative score). As presented in Table 1, ANOVA results showed that experiences of IPV were differentially distributed across monthly income, smoking status, and drinking status. In the post hoc tests, we found that the higher monthly income group (6001 RMB and above) had significantly higher IPV scores than the lower monthly income group (3000 RMB and below) (all *ps* < 0.05). Similarly, compared to transgender women without smoking or drinking, those with smoking or drinking had higher IPV scores (*ps* < 0.05).

Furthermore, among the participants, a substantial proportion reported experiencing IPV during their lifetime (83.7%). The most common forms of IPV were psychological IPV (76.9%) and transgender-specific IPV (76.5%), followed by physical IPV (66.7%) and sexual IPV (60.2%). To identify factors associated with IPV, we further conducted univariate logistic regression analyses using IPV experience as a categorical outcome variable. The results indicated that monthly income, length of residence in Shenyang, and drinking status were significantly associated with IPV.

Compared to the lowest income group (≤ 3000 RMB), those in the 6001–12,000 RMB group had higher odds of IPV (OR = 4.035, 95% CI = 1.518–10.724), and those in the ≧ 12,001 RMB group also had higher odds of IPV (OR = 8.421, 95% CI = 1.069 − 66.350). The 3001 − 6000 RMB group showed no significant association (*p* > 0.05). Estimates for some categories, particularly the highest income group, had wide confidence intervals and should be interpreted cautiously. Compared to local residents (reference group), those with 1 to 5 years of local residence exhibited a higher odds of IPV (OR = 2.766, 95% CI = 1.078 − 7.095). No significant association was found for those with less than 1 year or more than 5 years of local residence (*p* > 0.05). Additionally, when compared to non-drinkers, individuals with a history of alcohol consumption were found to have a higher odds of IPV (OR = 3.069, 95% CI = 1.535 − 6.135). Smoking status was associated with continuous IPV scores but not with dichotomous IPV exposure, which may reflect the loss of information after dichotomization.

The results of Table 2 reveal that transgender women with IPV experiences reported higher depression and anxiety than those without IPV experiences.

### Network structure

**Fig. 1:**
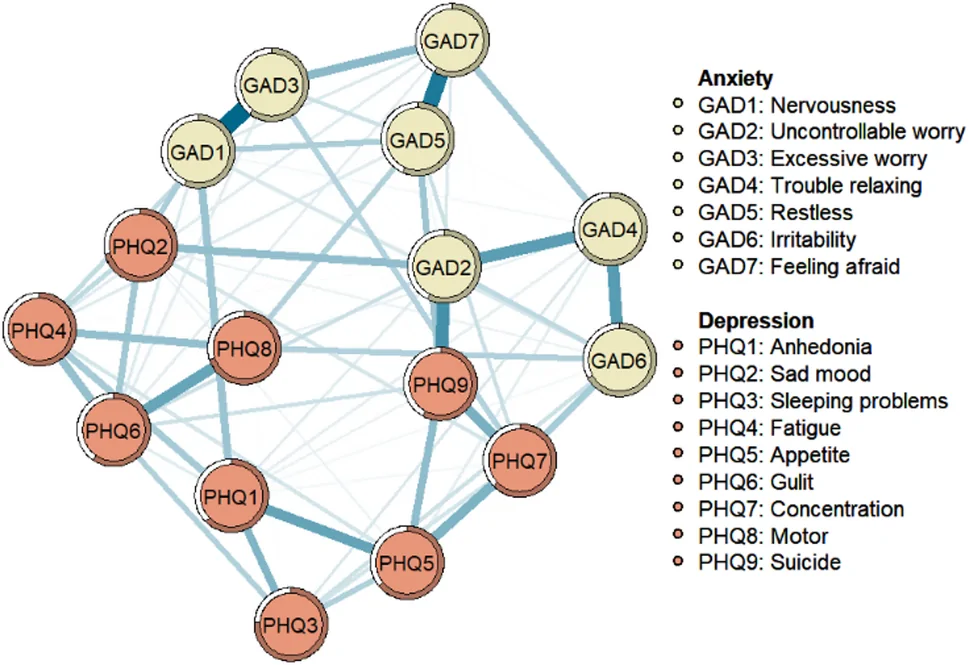
Network structure of depression and anxiety symptoms among Chinese transgender women (*n* = 264). Nodes are colored according to their clusters, with red nodes indicating depressive symptoms (PHQ-9) and yellow nodes indicating anxiety symptoms (GAD-7). Edges represent regularized partial correlations between symptoms. Thicker edges indicate stronger positive partial correlations, whereas thinner edges indicate weaker positive partial correlations

The network of depression and anxiety symptoms among transgender women is shown in Fig. 1. The ring pie chart around each node shows the predictability of each symptom. The network consisted of 120 regularized partial correlations, with 76 edges showing positive associations. Correlation matrices are displayed in online supplemental Table S1. In the anxiety network, the strongest edges (in descending order) were GAD1 (nervousness) with GAD3 (excessive worry), GAD5 (restlessness) with GAD7 (feeling afraid), GAD4 (trouble relaxing) with GAD6 (irritability), and GAD2 (uncontrollable worry) with GAD4 (trouble relaxing). In the depression network, the strongest edges (in descending order) were PHQ1 (anhedonia) with PHQ5 (loss of appetite), PHQ6 (guilt) with PHQ8 (reduced movement), PHQ7 (trouble concentrating) with PHQ9 (suicide), PHQ5 (loss of appetite) with PHQ7 (trouble concentrating), and PHQ1 (anhedonia) with PHQ3 (sleep disturbance). In the combined depression and anxiety network, the strongest edge was GAD2 (uncontrollable worry) with PHQ9 (suicide).

**Fig. 2:**
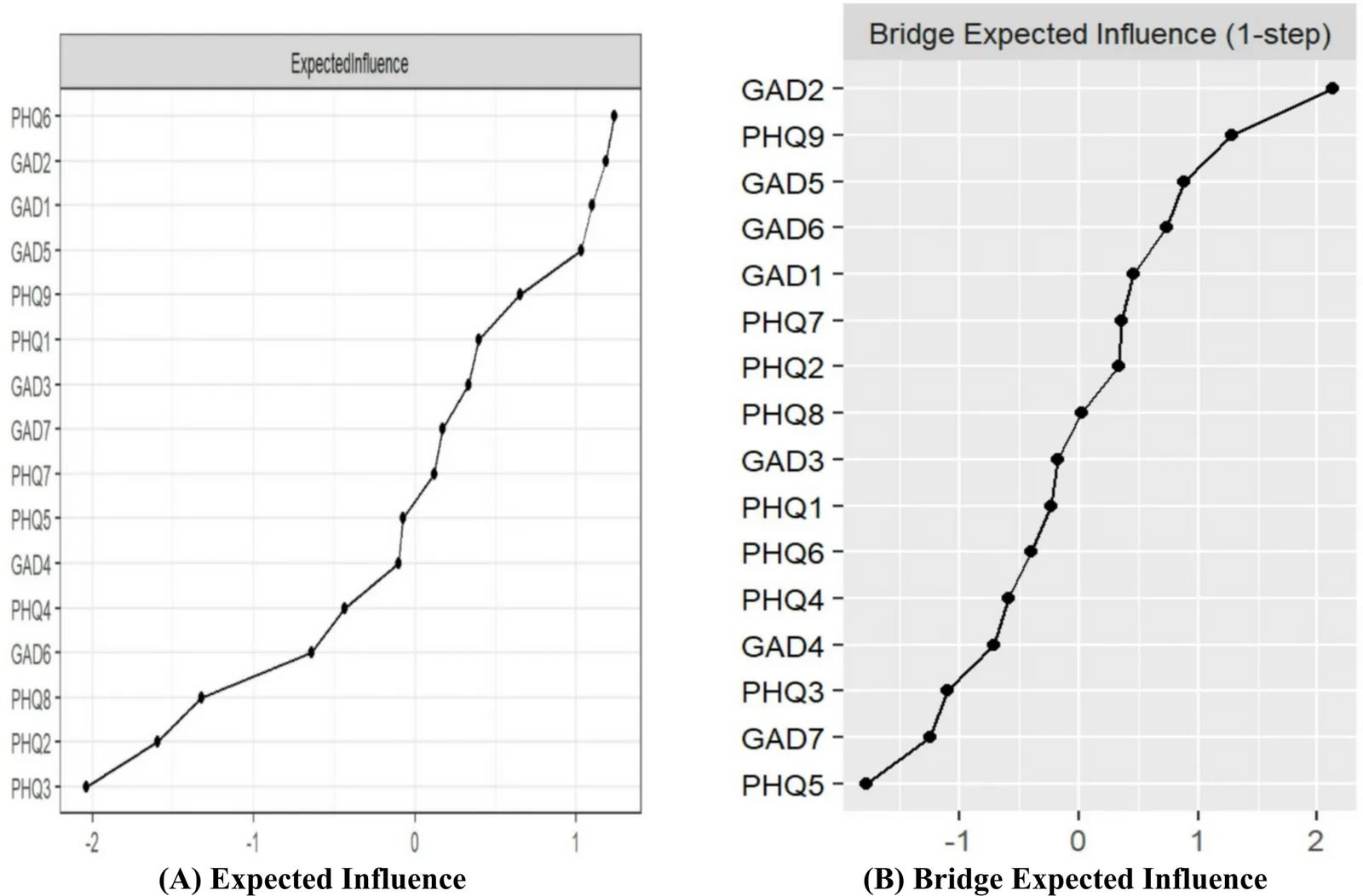
Centrality indices: expected influence (**A**) and bridge expected influence values (**B**)

The EI and bEI values of symptoms in the depression and anxiety network are shown in Fig. 2. The most central symptoms in the estimated depression–anxiety network were PHQ6 (guilt), GAD2 (uncontrollable worry), and GAD1 (nervousness) (Fig. 2A, left “part”). The main bridge symptoms between the depression and anxiety communities were GAD2 (uncontrollable worry), PHQ9 (suicidal ideation), and GAD5 (restlessness) (Fig. 2B, right “part”). These indices reflect statistical connectedness within the estimated network and should not be interpreted as evidence of causal influence.

**Fig. 3:**
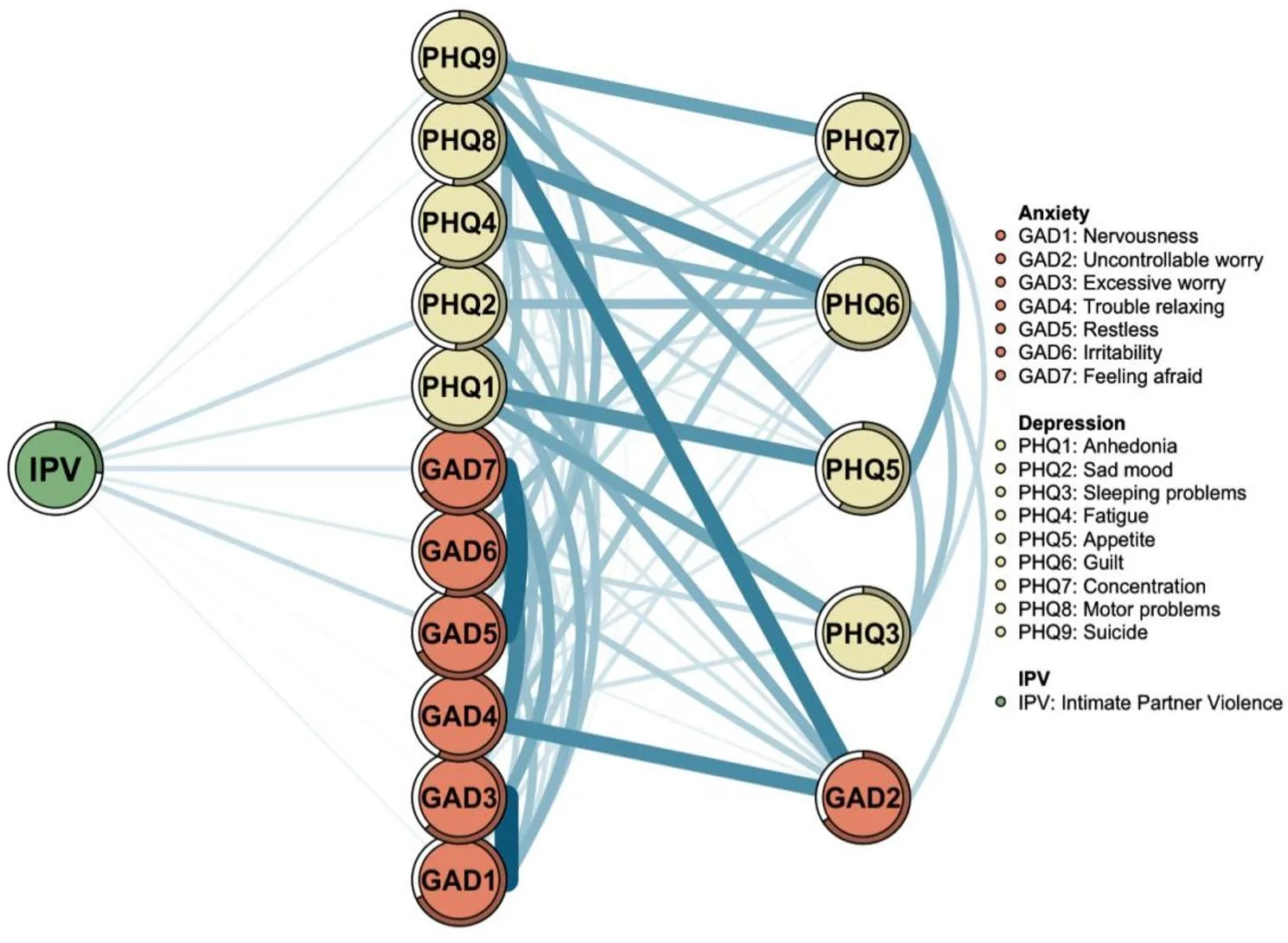
Flow network illustrating associations between intimate partner violence (IPV) and depressive and anxiety symptoms, adjusted for monthly income, length of local residence, smoking status, and drinking status. Nodes on the right represent individual depression and anxiety symptoms. The node on the left represents IPV, treated as an external binary exposure variable (not a psychiatric symptom). Edges represent partial correlations between IPV and each symptom. Thicker edges indicate stronger positive associations

The flow network of IPV with depressive and anxiety symptoms is shown in Fig. 3. The partial correlations between IPV and each symptom were estimated while controlling for monthly income, length of local residence, smoking, and drinking. Under these adjusted models, the strongest edges between IPV and depressive and anxiety symptoms included GAD5 (restlessness) and PHQ2 (sad mood).

### Network stability

**Fig. 4:**
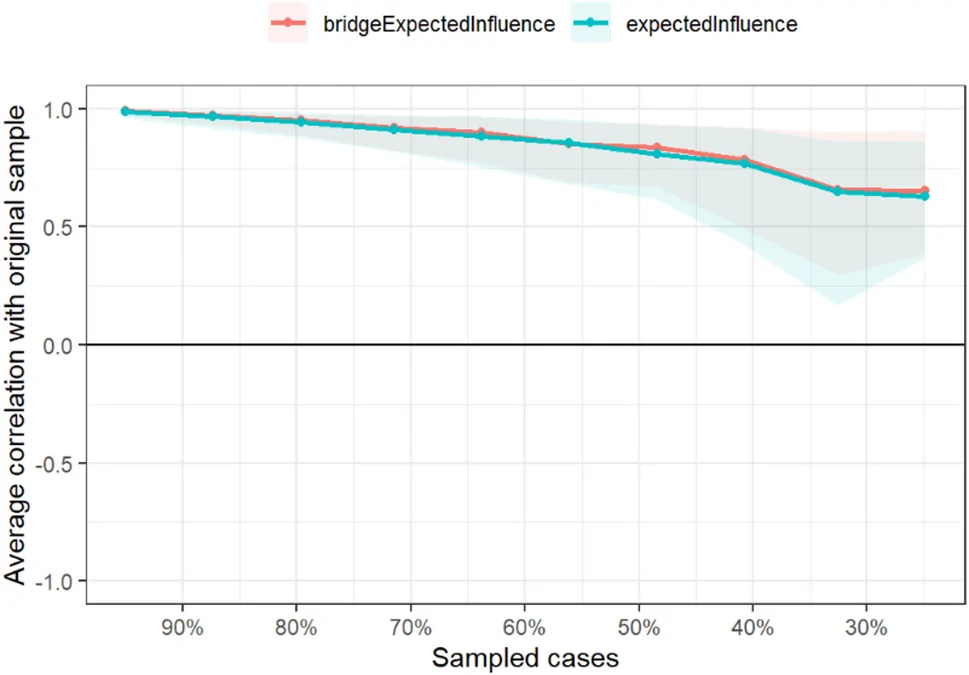
The stability of centrality and bridge centrality indices according to a case-dropping bootstrap method

The EI and bEI indices showed acceptable stability. The CS-coefficient was 0.439 for EI and 0.362 for bEI (Fig. 4), indicating that the centrality rankings were moderately stable under case-dropping bootstrap procedures. The bootstrapped stability test for EI (Figure S1) further confirmed that the central symptoms had significantly higher expected influence than most other nodes. Nevertheless, because the bridge centrality stability was only modest, the rank order of bridge symptoms should be interpreted cautiously.

### Comparison of the network structure between the IPV and non-IPV groups

The network structures generated from the two groups are shown in Figure S2 and S3. Comparisons of networks generated for IPV group versus non-IPV group revealed no significant differences in network structure (M = 0.431, *p* = 0.064) and global strength (IPV group: 7.349 vs. non-IPV group: 7.469; S = 0.121, *p* = 0.801).

## Discussion

The substantive contribution of this study is not the application of network analysis alone, but the identification of symptom-level mental health correlates of IPV in a highly marginalized population for whom empirical mental health data remain scarce in China. To the best of our knowledge, this is the first study to use network analysis to examine the symptoms of depression and anxiety among Chinese transgender women and their associations with IPV. Notably, the lifetime IPV prevalence in our sample was 83.7%. In our network of depression and anxiety symptoms, the most central symptom was PHQ6 (guilt), followed by GAD2 (uncontrollable worry) and GAD1 (nervousness); these symptoms showed the strongest statistical connectedness with other depressive and anxiety symptoms in the estimated network. Furthermore, the bridge symptoms linking depression and anxiety communities were GAD2 (uncontrollable worry), PHQ9 (suicide), and GAD5 (restlessness). The strongest edges between IPV and depression and anxiety symptoms were GAD5 (restlessness) and PHQ2 (sad mood).

PHQ6 (guilt) was the most central symptom in our network of depression and anxiety symptoms. To our knowledge, this is the first report to highlight the centrality of guilt among Chinese transgender women. This finding is consistent with Cognitive Behavioral Therapy (CBT) principles, which emphasize the role of cognition—particularly negative thought patterns such as self-criticism, guilt, and feelings of worthlessness—in shaping emotions and maintaining depressed mood [34]. The salience of guilt among Chinese transgender women may be tentatively understood within the sociocultural context in which they are embedded [35]. In Chinese patriarchal culture, son preference is a widely observed social phenomenon, and parents expect to continue the bloodline through a male heir [36]. Previous research on Chinese transgender women has shown that parents often blame them for shirking the responsibility of producing offspring as a male heir [37]. Such a culturally specific form of blame could theoretically contribute to the salience of guilt in this population; however, given the cross-sectional nature of this study, the role of guilt as a potential intervention target remains to be validated by experimental research. Clinically, these preliminary findings suggest that it may be beneficial for practitioners to consider the role of experiences of guilt when assessing and treating Chinese transgender women’s depression and anxiety.

GAD2 (uncontrollable worry) and GAD1 (nervousness) were two other central symptoms in our study, consistent with several previous studies based on depression and anxiety network models in adolescents [38, 39] and adult psychiatric patients [40]. This pattern may be understood through the lens of minority stress theory. Minority stress refers to an additional, unique form of stress experienced by individuals with a minority status—stress that is not experienced by those without that status. For transgender women in China, these unique stressors are frequently linked to their gender minority identity and are associated with elevated levels of anxiety and depression. The prominent role of worry and nervousness in our study aligns with the Gender Minority Stress and Resilience Model [41, 42], which identifies three dimensions of minority stress: (a) distal stress—externally experienced events such as harassment or discrimination; (b) anticipatory stress—the accumulation of worries that something bad is about to happen, such as rejection anticipation; and (c) proximal stress—internalized stress, such as internalized transphobia [43]. Researchers have proposed that gender minority stress—including interpersonal victimization, internalized stigma, and rejection—contributes to higher levels of stress, which may in turn lead to anxiety and depressive symptoms [44, 45]. This is consistent with broader psychopathological perspectives suggesting that interpersonal and environmental stressors play a fundamental role in shaping the clinical presentation of vulnerable populations [46].

GAD2 (uncontrollable worry), PHQ9 (suicide ideation), and GAD5 (restlessness) emerged as bridge symptoms, suggesting that they may be statistically relevant to the co-occurrence of depressive and anxiety symptoms in this sample. These findings align with previous network studies in Chinese medical students [39], patients with major depressive disorder [47], and psychiatric caregivers [48]. Theoretically, these bridge symptoms may operate through distinct mechanisms. Uncontrollable worry (GAD2) may represent a cognitive pathway whereby persistent negative expectations about future threats (e.g., rejection, discrimination) erode hope and generate depressive symptoms [49]. Suicidal ideation (PHQ9) may reflect the severe end of the comorbid spectrum, as the co-occurrence of anxiety and depression substantially increases suicide risk [50]. Restlessness (GAD5) may serve as a somatic bridge, linking the hyperarousal of anxiety with the agitated state often seen in mixed depressive episodes [51]. Together, these findings suggest that comorbidity is actively maintained by specific symptoms that cross diagnostic boundaries. Clinically, these bridge symptoms may warrant attention during assessment and treatment planning, particularly when depressive and anxiety symptoms co-occur. However, longitudinal and intervention studies are needed before these symptoms can be considered validated treatment targets.

Our study identified a lifetime IPV prevalence of 83.7%, a figure that is comparable to the upper range reported in prior meta-analytic evidence on IPV among transgender populations [6]. This high estimate likely reflects our comprehensive assessment strategy, which captured multiple dimensions of violence, including psychological and emotional aggression—forms of abuse that are often more pervasive yet underreported compared to physical or sexual violence. Furthermore, such a high prevalence underscores the unique psychosocial stressors and structural vulnerabilities experienced by this marginalized community. These findings provide a crucial high-prevalence context that is essential for interpreting both the subsequent network structure and the dense connectivity between psychological symptoms observed in our analysis.

IPV has been widely reported to have a positive relationship with depression and anxiety [9]. In our study, GAD5 (restlessness) and PHQ2 (sad mood) had the strongest positive associations with IPV among all symptoms examined, indicating that these key symptoms may be closely intertwined with IPV experience in transgender women. GAD5 (restlessness), an essential diagnostic symptom of generalized anxiety disorder [52], showed the strongest association with IPV. In the context of IPV, restlessness may co-occur with heightened arousal and hypervigilance, reflecting the interconnected nature of anxiety symptoms and IPV exposure. Notably, GAD5 serves a dual role in our network analysis: it functions both as a bridge symptom linking depression and anxiety and as the symptom most strongly connected to IPV. This dual role suggests that restlessness may be a particularly important symptom to monitor clinically, although longitudinal research is needed to determine whether targeting restlessness could improve IPV-related outcomes and overall psychological distress. PHQ2 (sad mood) had the second strongest association with IPV. From a dialectical behavior therapy perspective, sad mood (a negative emotion) may serve as an escape motivation that increases risk for future suicide behaviors, anxiety symptoms, and other behavioral problems [53]. Taken together, these findings tentatively suggest that anxiety symptoms (exemplified by restlessness) may be more closely associated with IPV than depressive symptoms in this population. However, this finding should be considered preliminary and remains a hypothesis requiring further validation.

Importantly, our findings should not be interpreted as suggesting a causal pathway from depression and anxiety symptoms to IPV victimization. IPV was treated as an external binary exposure variable rather than a symptom node within the depression–anxiety network. The flow network was used solely to visualize partial correlations between IPV and each symptom, without implying that IPV belongs to the same symptom network or occupies a symmetric position. Instead, IPV is understood as a major social and interpersonal risk factor for poor mental health among transgender women. A substantial body of literature has demonstrated that IPV exposure can contribute to the development and exacerbation of depression, anxiety, post-traumatic stress, suicidal ideation, and other adverse mental health outcomes. Thus, the observed associations are best interpreted as concurrent correlations consistent with a directional pathway from IPV to psychological distress, although the cross-sectional design precludes causal claims about either direction. Critically, responsibility for IPV always lies with the perpetrator, not the victim.

The network comparison revealed no significant differences in global strength or structure (*p* > 0.05) between Chinese transgender women who experienced IPV and those who did not. These findings suggest a dissociation between symptom severity and network topology; specifically, while IPV exposure may elevate the mean levels of individual symptoms, it does not necessarily reconfigure the inter-item connectivity patterns. Indeed, Chinese transgender women with IPV scored significantly higher on all anxiety and depression items. As such, IPV experience may be better understood as being associated with increased symptom severity rather than a symptom of depression or anxiety itself. Nonetheless, these null findings should be interpreted with caution, as empirical comparisons in this specific population remain limited.

Several limitations should be considered when interpreting the findings of this study. First, the cross-sectional, self-report design precludes causal inferences and may introduce common method variance as well as reporting biases, particularly given the sensitive nature of IPV and mental health symptoms. Unmeasured confounders, such as minority stress, stigma, trauma history, substance use, or social support could influence the observed associations. Second, the relatively modest sample size limited our ability to conduct detailed subgroup analyses. Third, although the stability of bridge centrality in our sample was acceptable, it was not particularly high. Therefore, while our results suggest that GAD2, PHQ9, and GAD5 may serve as bridge symptoms in this population, the rank order of their centrality should not be overinterpreted. Replication in larger, independent samples is needed to confirm which specific symptoms are the most critical bridges between depression and anxiety in Chinese transgender women. Fourth, the IPV and non-IPV subgroups were imbalanced in size. While increasing permutations improved the numerical stability of the permutation test, it could not overcome the limited statistical power due to the small non-IPV subgroup. Hence, the null NCT findings should be considered exploratory. Fifth, participants were recruited through snowball sampling from a community-based NGO in a single Chinese city, which may introduce selection bias; thus, findings may not be fully generalizable to transgender women in other regions or those not engaged with community networks. Sixth, individuals with severe mental illness were excluded, potentially reducing symptom variability and limiting generalizability to more clinically severe populations. Finally, the high IPV prevalence (83.7%) should be interpreted with caution, as it may reflect our comprehensive assessment strategy (which included psychological aggression) and community-based recruitment. Additionally, the dichotomous classification of IPV may reduce variability and omit information on violence frequency or severity, which could potentially influence the observed network associations. Despite these limitations, this study provides the first network analysis of depression, anxiety, and IPV among Chinese transgender women.

These limitations notwithstanding, our findings carry important public health implications. From a public health perspective, addressing IPV as a social determinant of mental health warrants further investigation—a need that is particularly salient in the Chinese context. Due to the increased risk of HIV among sexual and gender minority populations, transgender women have received disproportionate attention in HIV prevention and public health interventions [54], yet their mental health needs remain largely overshadowed. With an estimated 400,000 transgender people in China [55] and a growing population due to advances in HIV treatment and prevention [56], the demand for mental health care will likely intensify. However, the healthcare system faces persistent structural barriers—including lack of provider training, cisgender-centric service design, and stigma and discrimination—that may prevent these needs from being met. Accordingly, comprehensive support strategies for Chinese transgender women should explore the integration of symptom-focused mental health interventions (e.g., targeting central and bridge symptoms) with broader systemic efforts, including routine IPV screening, trauma-informed care, community-based support programs, and structural interventions to prevent IPV. Addressing IPV as a social determinant of mental health—alongside the existing HIV-focused framework—may represent a more integrated approach to reducing mental health disparities in this population.

## Conclusion

In conclusion, this exploratory network analysis of depression, anxiety, and IPV among Chinese transgender women identified guilt, uncontrollable worry, and nervousness as central symptoms, and uncontrollable worry, suicidal ideation, and restlessness as bridge symptoms. Restlessness and sad mood showed the strongest associations with IPV exposure in the flow network. IPV exposure was associated with higher symptom severity, but no significant network structural differences were detected between IPV and non-IPV groups. These findings should be interpreted as hypothesis-generating and require replication in larger, longitudinal, and more representative samples. Clinically and programmatically, the identified symptoms may help inform culturally sensitive assessment and mental health support, while IPV prevention requires survivor-centered, trauma-informed, and structural interventions.

## Supplementary Information

Supplementary Material 1.

## Data Availability

Data supporting the results of this study are available on request from the corresponding author.

## Abbreviations

GAD-7: Generalized Anxiety Disorder Questionnaire
PHQ-9: Patient Health Questionnaire
IPV: Intimate partner violence
