# Supplementary material for: Network analysis of intimate partner violence and depression–anxiety symptoms in Chinese transgender women
Source: BMC Public Health. 2026 Jul 27;26:3030. doi: 10.1186/s12889-026-28745-8 (PMC13685145; doi:10.1186/s12889-026-28745-8)
Supplement: Supplementary file 1 [file 12889_2026_28745_MOESM1_ESM.docx]

Table S1

Estimated edge weights among transgender women (n=264)

|  | GAD1 | GAD2 | GAD3 | GAD4 | GAD5 | GAD6 | GAD7 | PHQ1 | PHQ2 | PHQ3 | PHQ4 | PHQ5 | PHQ6 | PHQ7 | PHQ8 | PHQ9 |
| --- | --- | --- | --- | --- | --- | --- | --- | --- | --- | --- | --- | --- | --- | --- | --- | --- |
| GAD1 |  |  |  |  |  |  |  |  |  |  |  |  |  |  |  |  |
| GAD2 | 0.000 |  |  |  |  |  |  |  |  |  |  |  |  |  |  |  |
| GAD3 | 0.420 | 0.002 |  |  |  |  |  |  |  |  |  |  |  |  |  |  |
| GAD4 | 0.019 | 0.269 | 0.000 |  |  |  |  |  |  |  |  |  |  |  |  |  |
| GAD5 | 0.136 | 0.024 | 0.084 | 0.010 |  |  |  |  |  |  |  |  |  |  |  |  |
| GAD6 | 0.065 | 0.105 | 0.000 | 0.275 | 0.000 |  |  |  |  |  |  |  |  |  |  |  |
| GAD7 | 0.058 | 0.063 | 0.192 | 0.150 | 0.373 | 0.000 |  |  |  |  |  |  |  |  |  |  |
| PHQ1 | 0.163 | 0.049 | 0.000 | 0.042 | 0.000 | 0.019 | 0.000 |  |  |  |  |  |  |  |  |  |
| PHQ2 | 0.138 | 0.178 | 0.001 | 0.000 | 0.000 | 0.035 | 0.005 | 0.000 |  |  |  |  |  |  |  |  |
| PHQ3 | 0.000 | 0.000 | 0.000 | 0.063 | 0.000 | 0.082 | 0.000 | 0.206 | 0.000 |  |  |  |  |  |  |  |
| PHQ4 | 0.000 | 0.000 | 0.098 | 0.014 | 0.055 | 0.000 | 0.052 | 0.144 | 0.104 | 0.000 |  |  |  |  |  |  |
| PHQ5 | 0.000 | 0.000 | 0.000 | 0.046 | 0.000 | 0.000 | 0.000 | 0.253 | 0.034 | 0.130 | 0.070 |  |  |  |  |  |
| PHQ6 | 0.060 | 0.086 | 0.046 | 0.000 | 0.010 | 0.000 | 0.046 | 0.054 | 0.151 | 0.129 | 0.173 | 0.000 |  |  |  |  |
| PHQ7 | 0.000 | 0.001 | 0.124 | 0.000 | 0.087 | 0.143 | 0.005 | 0.020 | 0.069 | 0.074 | 0.000 | 0.213 | 0.000 |  |  |  |
| PHQ8 | 0.014 | 0.000 | 0.000 | 0.038 | 0.126 | 0.127 | 0.006 | 0.002 | 0.032 | 0.008 | 0.175 | 0.000 | 0.250 | 0.000 |  |  |
| PHQ9 | 0.000 | 0.306 | 0.012 | 0.000 | 0.158 | 0.010 | 0.009 | 0.035 | 0.000 | 0.000 | 0.000 | 0.185 | 0.084 | 0.219 | 0.000 |  |

Figure S1. Bootstrapped stability test for ‘expected influence’.

Figure S2. Network structure of depressive–anxiety symptoms among the non- IPV group Expected influence of the non-IPV group.

Figure S3. Network structure of depressive–anxiety symptoms among the IPV group and Expected influence of the IPV group.
